# Suppressing Glucose Transporter Gene Expression in Schistosomes Impairs Parasite Feeding and Decreases Survival in the Mammalian Host

**DOI:** 10.1371/journal.ppat.1000932

**Published:** 2010-06-03

**Authors:** Greice Krautz-Peterson, Mariana Simoes, Zahra Faghiri, David Ndegwa, Guilherme Oliveira, Charles B. Shoemaker, Patrick J. Skelly

**Affiliations:** 1 Molecular Helminthology Laboratory, Division of Infectious Diseases, Department of Biomedical Sciences, Tufts University, Cummings School of Veterinary Medicine, Grafton, Massachusetts, United States of America; 2 Laboratory of Cellular and Molecular Parasitology and CEBio, Instituto René Rachou - FIOCRUZ, Belo Horizonte, MG, Brazil; 3 Instituto Nacional de Ciência e Tecnologia em Doenças Tropicais, Salvador, BA, Brazil; George Washington University Medical Center, United States of America

## Abstract

Adult schistosomes live in the host's bloodstream where they import nutrients such as glucose across their body surface (the tegument). The parasite tegument is an unusual structure since it is enclosed not by the typical one but by two closely apposed lipid bilayers. Within the tegument two glucose importing proteins have been identified; these are schistosome glucose transporter (SGTP) 1 and 4. SGTP4 is present in the host interactive, apical tegumental membranes, while SGTP1 is found in the tegumental basal membrane (as well as in internal tissues). The SGTPs act by facilitated diffusion. To examine the importance of these proteins for the parasites, RNAi was employed to knock down expression of both SGTP genes in the schistosomula and adult worm life stages. Both qRT-PCR and western blotting analysis confirmed successful gene suppression. It was found that SGTP1 or SGTP4-suppressed parasites exhibit an impaired ability to import glucose compared to control worms. In addition, parasites with both SGTP1 and SGTP4 simultaneously suppressed showed a further reduction in capacity to import glucose compared to parasites with a single suppressed SGTP gene. Despite this debility, all suppressed parasites exhibited no phenotypic distinction compared to controls when cultured in rich medium. Following prolonged incubation in glucose-depleted medium however, significantly fewer SGTP-suppressed parasites survived. Finally, SGTP-suppressed parasites showed decreased viability *in vivo* following infection of experimental animals. These findings provide direct evidence for the importance of SGTP1 and SGTP4 for schistosomes in importing exogenous glucose and show that these proteins are important for normal parasite development in the mammalian host.

## Author Summary

Schistosomes are parasitic worms that live in the blood streams of ∼200 million people globally. They import glucose from host blood directly across their skin (the tegument). In the tegument the parasites possess glucose transporter proteins designated SGTP1 and SGTP4. SGTP4 sits on the outermost tegumental membranes while SGTP1 sits in the tegumental basal membrane (and on internal tissues). We have long hypothesized that SGTPs are involved in taking in glucose from the host but until the advent of advanced molecular technologies for use with schistosomes (notably RNA interference), we could not test this fundamental notion. In this work we employed RNAi to suppress expression of both SGTP genes in schistosomes. In support of our hypothesis, we find that SGTP1 or SGTP4-suppressed schistosomes do exhibit an impaired ability to import glucose compared to control worms and that this effect is compounded by suppression of both genes simultaneously. When suppressed parasites are cultured in glucose-depleted medium fewer of them survive. In addition, suppressed parasites showed decreased viability in experimental animals. These findings provide direct evidence of the importance of these tegumental transporters for schistosome feeding and show that these SGTPs are important for normal parasite development in the mammalian host.

## Introduction

*Schistosoma mansoni* is a parasitic platyhelminth that causes the chronic, often debilitating disease, schistosomiasis affecting several hundred million people globally. Infection is initiated following skin penetration by larval parasites called cercariae which rapidly adapt to the intra-mammalian environment in a process called cercarial transformation. These transformed juvenile parasites are now called schistosomula and they move from the epidermal tissues into the blood stream where they mature. Adult worms reside in the mesenteric veins of their mammalian hosts, where they are generally found as male-female pairs.

The entire worm is surrounded by a continuous cytoplasmic unit, or syncytium, called the tegument. The host interactive surface of the tegument is unusual in that it consists of two tightly apposed, lipid bilayer membranes that are highly invaginated. The internal, basal membrane of the tegument consists of a normal (trilaminate) lipid bilayer containing many invaginations. This bilayer extends periodically beneath the underlying muscle to enclose areas called “cell bodies” (or cytons) which contain nuclei and protein synthetic machinery [1].

Adult worms use large quantities of host glucose; they are reported to consume the equivalent of their dry weight in glucose every 5 hours [2]. While the adults possess a functional gut, they have been shown to take up glucose directly across their external body surface by facilitated diffusion [3], [4]. Three glucose transporter mRNAs were originally identified from *Schistosoma mansoni* and these were designated schistosome glucose transporter protein (SGTP) 1, 2 and 4 [5]. Only SGTP1 and SGTP4 displayed glucose transport activity when expressed in *Xenopus laevis* oocytes. In the *Xenopus* uptake assay, both proteins functioned as typical facilitated diffusion glucose transporters, exhibiting glucose stereospecificity, relaxed specificity for other hexoses, sodium independence and marked inhibition by cytochalasin B [5].

Immunolocalization of SGTP1 and SGTP4 revealed that both of these proteins are localized in the tegument of schistosomula and adult worms [6]. SGTP4 appears to be localized uniquely to the tegument, while SGTP1 can also be detected within the body of the worm, particularly in muscle [6]. The presence of facilitated diffusion transporters in the tegument implies that schistosomes have the capacity to take up glucose by passive diffusion. Localization of the SGTPs by immuno-electron microscopy reveals that SGTP4 is present predominantly or exclusively within the apical membranes, while tegumental SGTP1 is found only within the basal membrane [7], [8]. This asymmetrical localization of the two SGTPs in the tegument suggests that the host interactive protein (SGTP4), acts to import sugar from the bloodstream into the tegument and that SGTP1 acts to transport some portion of this sugar to underlying tissues. The *K*m for glucose transport by the apical tegumental membrane transporter SGTP4 is greater than that of the basal transporter SGTP1 (in *Xenopus* oocytes) [5]. This should give an advantage to the basal membrane transporter to associate with any free glucose that is not utilized in the tegumental syncytium so that it can be moved more deeply into the body of the worm.

We have long hypothesized that the SGTPs function to transport exogenous glucose across the tegumental membranes and into body of the worms [9]. However, until the advent of RNAi methods for use with schistosomes, we could not effectively test this fundamental notion. In this work, we show that suppressing SGTP gene expression using RNAi does impair schistosome glucose uptake capabilities and can debilitate the parasites *in vitro* and *in vivo*.

## Results

### The *Schistosoma mansoni* genome contains four facilitated glucose transporter genes

The availability of a nearly complete draft of the *S. mansoni* genome [10] permits a careful bioinformatic analysis for facilitated glucose transport protein genes and this identifies a total of four SGTP genes. In addition to the three genes previously identified, another facilitated glucose transporter homolog can now be identified. The gene, which we designate SGTP3, is currently identified as hypothetical protein Smp_127200. Searches of dbEST reveal that ESTs exist for all four SGTP genes demonstrating that these genes are expressed in mammalian stage schistosomes. Because SGTP1 and SGTP4 are clearly demonstrated to be expressed in the adult tegument and appear to be the predominant facilitated glucose transporters in adult *S. mansoni*, we focused our RNAi studies on these two genes [6].

### RNAi-induced knockdown of SGTP1 and SGTP4 gene expression in schistosomula and adult worms

**Figure 1:**
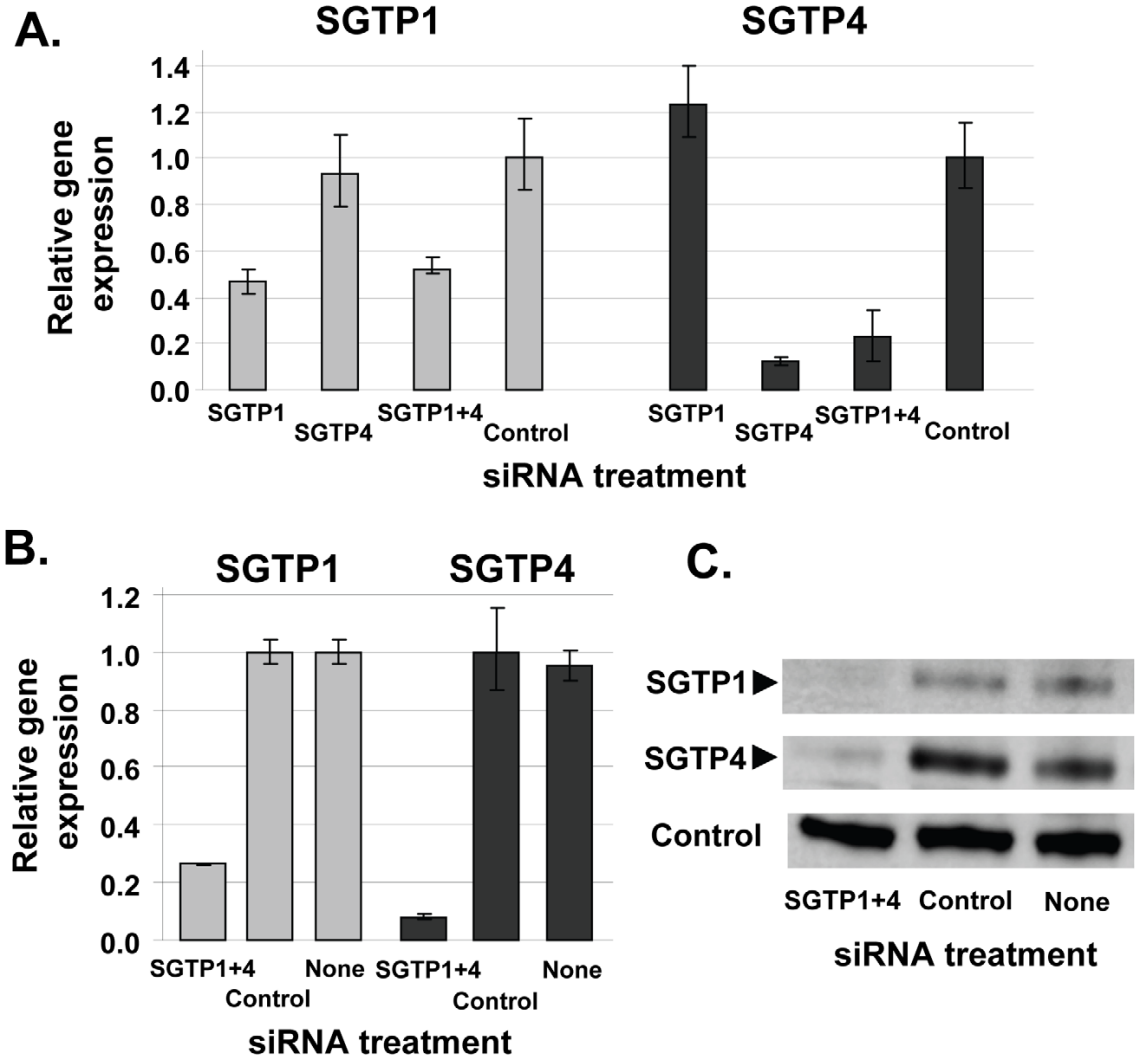
Schistosome glucose transporter protein (SGTP) gene expression analysis. A. Relative SGTP1 (grey bars) and SGTP4 (black bars) gene expression (mean ± SE) in schistosomula 7 days after treatment with control or SGTP siRNA. “SGTP1+4” indicates parasites treated with both SGTP1 and SGTP4 siRNAs. B. Relative SGTP1 (grey bars) and SGTP4 (black bars) gene expression (mean ± SE) in adult parasites 14 days after treatment with the indicated siRNA. C. Protein levels in adult worm extracts obtained 14 days following treatment with the indicated siRNA. Western blot results are shown for SGTP1 protein (top panel), SGTP4 (middle panel) and a control protein (SPRM1hc, bottom panel). The arrowheads indicate the diminished level of SGTP proteins seen in the left lanes.

To determine whether SGTP1 and SGTP4 are amenable to gene silencing in schistosomula via the RNAi pathway, parasites were treated with two siRNAs spanning distinct positions for each target. All siRNAs were effective and showed comparable knockdown for each target (not shown). One of each target-specific siRNA was then selected for all subsequent experiments: *SGTP1siRNA1* for SGTP1 and *SGTP4siRNA1* for SGTP4. Parasites were electroporated with *SGTP1siRNA1*, *SGTP4siRNA1*, or a mix or both siRNAs. Control parasites were treated with an irrelevant siRNA or were not exposed to siRNA at all. Parasites were then cultured for 14 days in Basch medium before being harvested for gene expression analysis. Figure 1 shows that the transcript levels of both targets were substantially reduced when parasites were treated with each siRNA separately or in combination, compared to controls (Figure 1A). Gene knockdown is specific; siRNAs targeting SGTP1 have no effect on SGTP4 expression levels and *vice versa*. The reduction in transcript levels was more striking for SGTP4 (∼85%) than for SGTP1 (∼55%). Schistosomula treated with an siRNA targeting SGTP1 alone or with a mix of siRNAs targeting both SGTP1 and SGTP4 exhibited a similar decrease in SGTP1 gene expression. Likewise, parasites treated with an siRNA targeting SGTP4 alone or with a mix of siRNAs targeting both SGTPs exhibited a similar decrease in SGTP4 gene expression, as depicted in Figure 1.

To monitor SGTP gene suppression in adults, six week old worms were recovered from infected mice and electroporated with SGTP1 plus SGTP4 siRNAs. Transcript levels were measured 14 days after siRNA treatment by qRT-PCR and the results are shown in Figure 1B. About 70% suppression of SGTP1 and 90% of SGTP4 was observed in adult parasites following treatment, compared to control parasites electroporated with an irrelevant siRNA or parasites electroporated in the absence of siRNA. Western blotting analysis was undertaken in order to assess the impact of gene suppression on target protein levels. Figure 1C shows that the suppression of both targets resulted in a substantial diminution of SGTP1 (top panel) and SGTP4 (middle panel) protein levels in these parasites. In contrast, both proteins were easily detected in extracts of control parasites. In the bottom panel, the amino acid permease control protein SPRM1hc was detected in all extracts, demonstrating that comparable levels of protein were present in each lane.

### Effect of SGTP1 and SGTP4 gene suppression on glucose import

**Figure 2:**
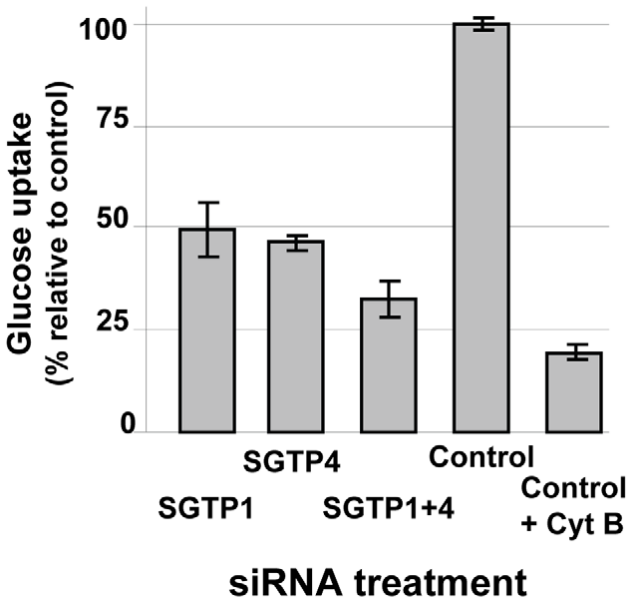
Glucose uptake analysis in schistosomes treated with SGTP siRNA. Relative uptake of 2-deoxyglucose (mean ± SE) into schistosomula 14 days after treatment with the indicated siRNA. An additional control group was treated with the glucose transport inhibitor, cytochalasin B (control +CytB, right bar).

The glucose uptake capacity of SGTP-suppressed schistosomula versus controls was compared. As shown in Figure 2, parasites treated with SGTP1 or SGTP4 siRNAs had a significant (*P* = 0.0005) and similar (∼50%) reduction in glucose uptake capacity compared to the control group. Parasites treated with a mix of both SGTP siRNAs showed an even more pronounced reduction in glucose uptake (to ∼70%) and this decrease was significantly different from the values obtained using single SGTP-suppressed parasites (*P* = 0.003). In the presence of cytochalasin B, a glucose transporter inhibitor, the intake of glucose by control parasites was decreased further (to ∼80%, *P* = 0.008) (Figure 2).

### Effect of SGTP1 and SGTP4 gene suppression on parasite viability *in vitro* and *in vivo*

**Figure 3:**
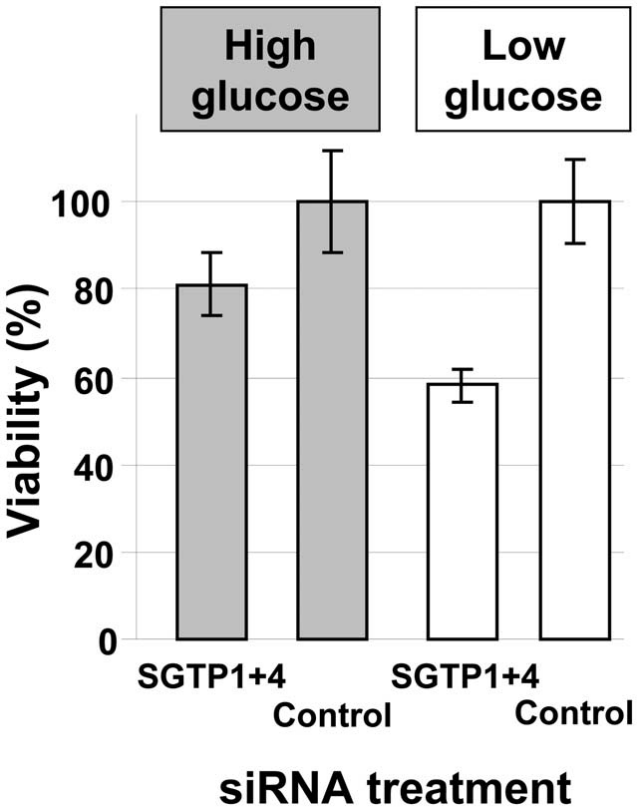
Relative viability of schistosomes treated with SGTP siRNA. Schistosomula (mean ± SD) were treated with the indicated siRNA and their viability was established following culture for 14 days in RPMI medium either containing high glucose (10 mM, grey bars) or low glucose (0.05 mM, white bars).

To determine whether SGTP suppression and the resulting decrease in glucose uptake capacity affected the phenotype of cultured schistosomula, parasite viability in culture was measured by Hoechst staining 14 days after siRNA treatment. Schistosomula were maintained either in complete RPMI (containing 10 mM glucose) or in glucose-depleted RPMI (containing 0.05 mM glucose). Figure 3 shows that suppressing the SGTP1 and SGTP4 genes did not significantly affect the viability of parasites kept in medium containing relatively high levels of glucose. However SGTP-suppressed parasites cultured in RPMI containing low glucose were significantly less viable (by >40%) than their control counterparts (*P* = 0.02). Parasites cultured in RPMI with no glucose do not survive beyond 48 hours. It is noteworthy that control parasites experience stress under low glucose conditions such that 62.4% ±5.6 of them remain viable after 14 days in culture (compared with 93.3% ±14.2 of control parasites cultured under high glucose conditions).

**Figure 4:**
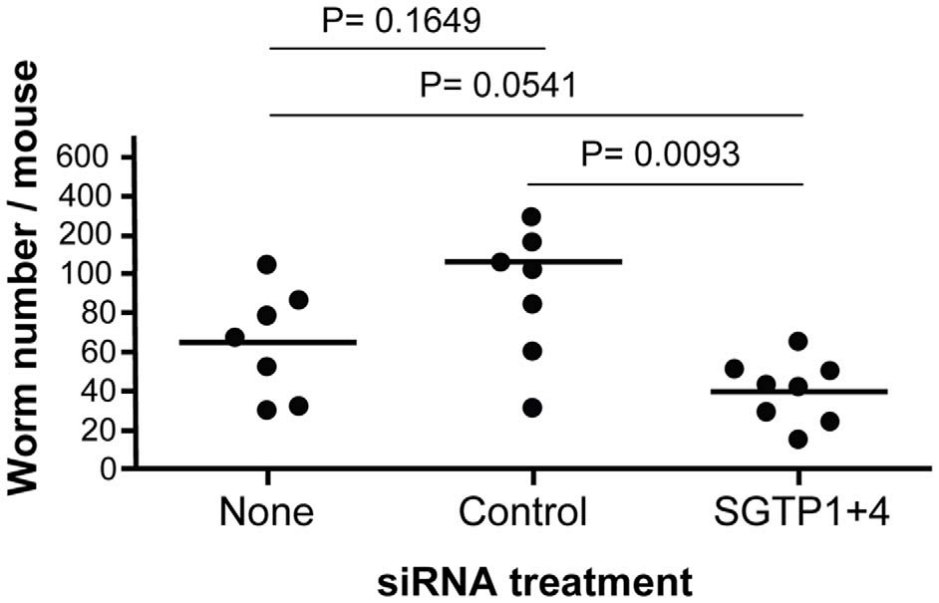
Schistosome survival *in vivo* in mice following treatment with SGTP siRNA. Schistosomula were treated with the indicated siRNA and used to infect mice. Worm numbers recovered from individual mice in each group are shown as dots. The lines indicate the mean for each group and P values are given at top.

To investigate whether RNAi-mediated gene silencing of SGTP1 and SGTP4 affects parasite viability *in vivo*, we infected groups of 7–8 mice with 1 day old control or SGTP1+ 4-suppressed schistosomula. Figure 4 shows the number of worms recovered from these mice 28 days after infection. There was a significant reduction in worm burden in the SGTP-suppressed group compared to either control group.

**Figure 5:**
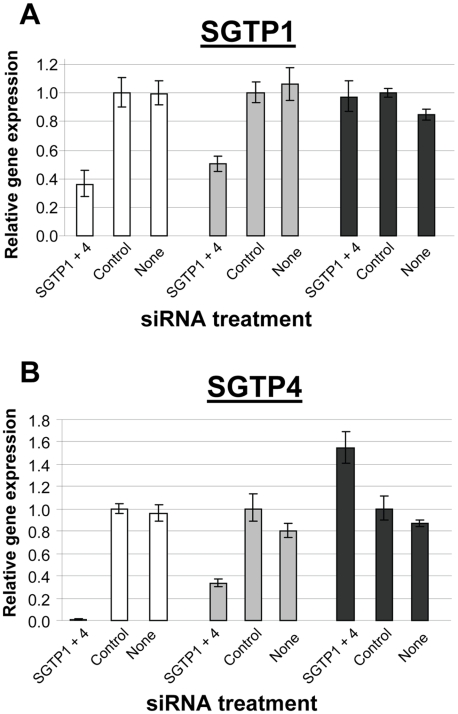
Expression of SGTP1 and SGTP4 (mean ± SE) in schistosomula at different times after treatment with the indicated siRNA. Parasites were either maintained in culture for 7 days after treatment (white bars) or for 4 week after treatment (grey bars), or were recovered from infected mice 4 weeks after treatment (black bars). A, SGTP1; B, SGTP4.

SGTP gene expression analysis was undertaken on the worms recovered from the infected mice and the data were compared to the gene expression pattern of suppressed and control schistosomula that had been maintained in culture. SGTP-suppressed parasites cultured for 7 days (white bars, Figure 5) exhibited ∼65% suppression of SGTP1 (Figure 5A) and close to 100% suppression of SGTP4 (figure 5B). After 28 days cultured *ex vivo* (grey bars, Figure 5), mRNA levels in the SGTP dsRNA-treated worms were rising but remained substantially lower than control levels (∼50% for SGTP1 (Figure 5A) and ∼70% for SGTP4 (Figure 5B)). In contrast, after 28 days *in vivo* (black bars, Figure 5), SGTP-suppressed parasites recovered from infected mice were no longer suppressed; SGTP transcript levels had returned to normal, or above normal, levels.

Essentially the same results were observed when schistosomula were treated with long dsRNAs specific for SGTP1 and SGTP4 by soaking. The level of gene suppression using this metholology was comparable to that reported above for parasites exposed to SGTP-specific siRNA by electroporation. Parasites treated with SGTP-specific, and control, long dsRNA were used to infect mice and were recovered by perfusion 28 days later. As for the siRNA work, significantly fewer worms were recovered from the SGTP-suppressed group versus controls in this experiment using long dsRNA (not shown). Finally, as seen with siRNA-treated parasites, recovered parasites were no longer suppressed (data not shown).

## Discussion

In this work we show that two *Schistosoma mansoni* glucose transporter (SGTP) genes, SGTP1 and SGTP4, are susceptible to suppression via RNAi. Of the two SGTPs targeted we find that SGTP4 is consistently better suppressed than SGTP1 using different siRNAs, long dsRNA and at two different life stages tested. This is consistent with the notion that genes expressed in schistosome tissues that are in direct contact with the environment (e.g. the tegument or the gut) are more efficiently suppressed by RNA interference compared to genes expressed in other tissues. SGTP4 is predominantly and perhaps exclusively expressed in the tegument [6], [7] whereas SGTP1 is additionally expressed in the internal tissues of the parasite, notably in the muscle [6], [8]. In the past we have noted that genes expressed predominantly or exclusively in the tegument (e.g. SmAQP) can be potently suppressed while those expressed both in the tegument and in internal tissues (e.g. SPRM1hc) are more poorly suppressed using the same protocols [11], [12], [13]. This may reflect differences in the ability of dsRNAs to enter internal tissues or to the differential expression of RNAi pathway components in different organs.

The level of SGTP4 gene suppression in schistosomula is ∼80%. This is the case when parasites are treated with dsRNA targeting SGTP4 alone or when treated with two siRNAs targeting SGTP4 and SGTP1. In a similar manner, the level of suppression of SGTP1 remains essentially the same when SGTP1 alone is targeted for suppression or when SGTP1 and SGTP4 are both targeted. These results support previous work [14] showing that more than one gene can be suppressed at one time in schistosomes. Our quantitative data show that the RNAi machinery is not saturated by multiple siRNAs targeting different mRNAs.

The level of inhibition of glucose uptake into SGTP1-suppressed parasites is comparable to that seen for SGTP4-suppressed parasites. When SGTP1 alone is suppressed, glucose should still be able to enter the parasite tegument freely via the outer tegumental membrane transporter SGTP4. However, the movement of imported glucose further into the body of the SGTP1-suppressed parasites would then be impaired since this transporter is present on the tegumental basal membranes and on the membranes of other internal tissues. The inability of imported glucose to be efficiently transported out of the tegument and into the deeper tissues using SGTP1 would increase tegumental glucose concentrations and likely impede the further import of glucose by facilitated diffusion from the external environment. This is reflected in lower radiolabeled glucose being taken in to the SGTP1-suppressed parasites compared to controls.

When SGTP4 alone is suppressed, less glucose should enter the worms across the tegument compared to controls but any glucose that does enter and that is not utilized within the tegument should be efficiently transported inward via SGTP1. This would promote further glucose diffusion into the parasites via residual tegumental SGTP4 transporters. Parasites with both SGTP1 and SGTP4 genes suppressed exhibit a significantly greater impairment of radiolabeled glucose uptake compared with parasites that have had just one of the transporter genes suppressed. This likely reflects both a lower level of glucose uptake into the tegument via SGTP4 and an impaired ability to move that glucose into the internal tissues via SGTP1. Note that the level of glucose uptake in the doubly suppressed parasites is higher than that seen in parasites treated with a chemical inhibiter of facilitated glucose transporter protein function - cytochalasin B. This compound has been shown to block SGTP1 and SGTP4 function since it inhibits radiolabeled glucose uptake into *Xenopus* oocytes that are expressing SGTP1 or SGTP4 [5]. The double SGTP knockdown parasites exhibited a higher glucose uptake (of ∼30% versus untreated controls) compared to parasites treated with cytochalasin B (whose uptake was ∼20% of untreated control parasites). Likely this reflects the high potency of cytochalasin B in almost completely shutting down all SGTP function. In contrast, RNAi leads to SGTP gene knockdown (but not gene knockout) and the presence of residual functional SGTP protein in the siRNA-treated groups does permit some label uptake. Residual protein includes any protein generated before siRNA administration as well as new protein derived from transcripts that survive the RNAi treatment. The diminished ability of SGTP-suppressed schistosomes to import glucose unequivocally demonstrates that these parasites do use both SGTP1 and SGTP4 to efficiently take in sugar. In a similar vein, earlier work reported that glucose uptake is impaired in schistosomes following exposure to SGTP antisense oligonucleotides [15]. However, this work noted non-specific effects with some oligonucleotides and considerable variability between treatments, making the data equivocal [15]. Previous work has demonstrated that SGTP1 is important for glucose uptake from the environment in the sporocyst life stage [16].

In order to establish whether the inability to import glucose by the SGTP-suppressed parasites had a detrimental impact on the worms, their viability was compared with that of control parasites *in vitro* and *in vivo*. Parasites in culture whose glucose transporter genes are suppressed show no significant phenotypic differences compared with controls, when they are maintained in medium with a high glucose concentration (10 mM) for up to 14 days. However, when these parasites are instead cultured in low glucose medium (0.05 mM) for 14 days, significantly fewer suppressed parasites survive compared with controls. This suggests that, in the sugar-poor environment, an impaired ability to import glucose upsets parasite metabolism and decreases viability. When SGTP-suppressed parasites infect mice, fewer of them survive to adulthood relative to controls. This is the case despite the fact that glucose concentrations in blood are high (∼5 mM). These data suggest that the parasites' glucose demands *in vivo* are higher than in culture and this likely reflects the need for parasites *in vivo* to generate more energy (through glucose catabolism) to allow them migrate through tissues, invade the vasculature and combat host immune effectors.

The level of RNAi-mediated target gene suppression diminishes with time in culture. After 4 weeks *in vitro* the level of suppression of SGTP1 is ∼50% compared with ∼65% at day 7 post treatment. For SGTP4 the suppression level at week 4 in culture is 70% compared with >95% at day 7. These data demonstrate that the RNAi effect remains substantial even after a month in culture. In contrast, equivalent parasites recovered from infected mice 4 weeks after RNAi treatment exhibit no remaining SGTP gene suppression. Those parasites that have survived *in vivo* have SGTP mRNA levels at or even above control levels. Similar variable outcomes of RNAi in schistosomes *ex vivo* compared to *in vivo* have been reported in other studies [17], [18]. One hypothesis is that RNAi is variably effective in different parasites and/or that different individuals in the treated parasite population received different amounts of siRNA. Those in which SGTP knockdown is least effective, or that received less dsRNA, survive because the expression of their SGTP genes is minimally impaired. Another hypothesis is that worms *in vivo* are more metabolically robust and this leads to a shorter half life of the dsRNA and/or its downstream effectors. In mammalian cells the longevity of the RNAi effect can depend on cell type: in non-dividing cells suppression can persist for several weeks whereas in rapidly dividing cells the effect may last only from 3 to 7 days. [19]. Schistosomes in culture appear quiescent; they do not develop as quickly and fully as do parasites in infected animals and this may contribute to the persistence of gene suppression observed in the cultured worms.

In summary, this work shows that by demonstrably suppressing glucose transporter gene expression in schistosomes using RNAi, parasite feeding is hindered and this can significantly lower parasite viability. These findings provide direct evidence for the importance of SGTP1 and SGTP4 for schistosomes in importing exogenous glucose and show that the proteins are important for normal parasite development within the mammalian host.

## Materials and Methods

### Ethics statement

Infection of mice with schistosome parasites was carried out following review and approval by the Institutional Animal Care and Use Committee of Tufts University or Instituto René Rachou - FIOCRUZ. The Tufts animal management program is accredited by the American Association for the Accreditation of Laboratory Animal Care, meets the National Institutes of Health standards as set forth in the “Guide for the Care and Use of Laboratory Animals” (National Academy Press, Washington DC, 1996), and accepts as mandatory the PHS “Policy on Humane Care and Use of Laboratory Animals by Awardee Institutions” and NIH “Principals for the Utilization and Care of Laboratory Animals Used in Testing, Research and Training”.

### Parasites

*Biomphalaria glabrata* snails infected with *S. mansoni* were obtained from Dr. Fred Lewis (Biomedical Research Institute, Rockville, MD). In some experiments parasites were obtained from snails infected at Instituto René Rachou - FIOCRUZ, Belo Horizonte, MG, Brazil. Schistosomula were prepared from cercariae released from infected snails and were cultured in Basch medium at 37°C, in an atmosphere of 5% CO_2_ as described [20]. Parasite viability was ≥90% at the beginning of each experiment as assessed by Hoechst staining [11]. In some experiments schistosomula were kept in complete RPMI medium which is RPMI supplemented with 10 mM Hepes, 2 mM glutamine, 5% fetal calf serum and antibiotics (100 U/ml penicillin and 100 µg/ml streptomycin). Adult worms were recovered by vascular perfusion from Balb/c mice that were infected with 125 cercariae, 6 weeks previously. Adult parasites were maintained in Basch medium for RNAi experiments.

### Treatment of parasites with double stranded RNA (dsRNA)

Schistosomula and adult worms were treated either with synthetic siRNAs (IDT, Coralville, IA) or with long dsRNAs specific for SGTP1 or SGTP4 (GenBank accession numbers L25065 and L25067, respectively). The siRNAs were designed with the help of the RNAi Design Tool at http://www.idtdna.com/Scitools/Applications/RNAi/RNAi.aspx. The siRNAs targeting SGTP1 are *SGTP1siRNA1*: 5′-GGAGCATTCAGTTGTGGTTGGGTTG-3′spanning the coding sequence at positions 229–254 and *SGTP1siRNA2*: 5′-ACATAAAGAAGCTGAGGCACGTAAA-3′ spanning the coding sequence at positions 647–672. The siRNAs targeting SGTP4 are *SGTP4siRNA1*: 5′-GAAATAGCTCCCTTATCTCTTCGTG -3′, which cover positions 447-472 of the coding sequence and *SGTP4siRNA2*: 5′-GTGACACCAAGTTTCTTATATGCTC-3′ which cover positions 186-211 of the coding sequence. The negative control siRNA (5′-CTTCCTCTCTTTCTCTCCCTTGTGA-3′) is the “DS Scrambled Neg” obtained from IDT, Inc. This sequence does not match any in the *S. mansoni* genome. Target-specific siRNA delivery to the parasites was performed by electroporation as described previously, using 2.5 µg/50 µl (2.8 µM) of each siRNA for schistosomula and 5 µg/50 µl (5.6 µM) for adults [21], [22].

Long dsRNA was prepared as described previously [21]. The primer sequences for preparing long dsRNA targeting SGTP1 are SGTP1-T7, 5′-ggtaatacgactcactatagggCTAATCGGATACAATCT-3′ and SGTP1-T3, 5′-ggaattaaccctcactaaagggAATGAAATACGAGAAA-3′ which spans the coding sequence at positions 79–514. The lower case sequences represent T7 or T3 RNA polymerase promoter sequences. SGTP4 long dsRNA was prepared as described [17]. A non-schistosome derived long dsRNA used as an irrelevant control was generated from the yeast expression plasmid pPIC9K, as described earlier [17]. Long dsRNA was delivered to the parasites by soaking cultured schistosomula overnight with 50 µg/ml of irrelevant or SGTP-specific long dsRNA [21], [22]. Gene suppression was assessed post-treatment by comparing mRNA and protein levels in target versus control groups.

### SGTP gene expression analysis

The levels of expression of SGTP1 and SGTP4 genes in schistosomula and adult worm pairs treated with gene-specific dsRNA were measured by quantitative real time PCR (qRT-PCR), using custom TaqMan gene expression systems from Applied Biosystems (Foster City, CA). The procedure, involving total RNA extraction and quantitative real time PCR, has been described [21]. The following primers and probe were selected to detect SGTP1: *SGTP1 forward*, 5′-CTGCAGCTTATTCACTGAGTCAATC- 3′; *SGTP1 reverse*, 5′-CCACCGATGTTTTTCTGTATAACAGGAT-3′ and *SGTP1 probe*, 5′-FAM- TCAATGGTTATCCAATCTAATTGT- 3′. To detect SGTP4 expression, the following primers and probe were used: *SGTP4 forward* 5′-AGCCAAGGAGTTAACTTATTATGCAATTTATTG 3′-; *SGTP4 reverse*, 5′- TCCAACAGATAATAACGATAACTAAAAATGGTAAGAA-3′ and *SGTP4 probe*, 5′-FAM- CAATGGCATCATTAATGC- 3′. Alpha tubulin was used as the endogenous control gene for relative quantification employing the ΔΔ*C*t method [23]. Results were graphed as gene expression level relative to the group treated with control irrelevant dsRNA.

### SGTP expression: western blotting analysis

Parasite lysates were prepared by adding 50 µl of ice cold cell disruption buffer (PARIS Kit, Ambion, TX) followed by incubation for 30 minutes on ice. The protein content in each extract was estimated using the BCA Protein Assay Kit (Pierce, IL) according to the manufacturer's instructions. Soluble protein (5 µg in 20 µl SDS-PAGE sample buffer) was subjected to SDS-PAGE under reducing conditions, blotted onto PVDF membrane and blocked using detector block solution (KPL, Inc.) for 1 h at room temperature. The membrane was then probed overnight at 4°C with affinity purified rabbit anti-SGTP1 or anti-SGTP4 serum at 1∶500 [5] or antibody directed against a control schistosome protein (SPRM1hc) [13]. Bound primary antibody was detected using goat anti-rabbit IgG conjugated to horseradish peroxidase (Invitrogen, Inc.), diluted 1∶5000, followed by incubation with the chemiluminescent substrate LumiGLO (KPL, Inc.) and the membrane was exposed to X-ray film. The same membrane was probed three times to detect SGTP1, SGTP4 and the loading control protein, SPRM1hc. For each re-use, the membrane was first incubated for 30 min at room temperature with 2% SDS and 0.7% β-mercaptoethanol to strip bound antibody and was then washed in phosphate buffered saline twice for 30 min each.

### Parasite viability measurement

To evaluate if SGTP1 and SGTP4 gene knockdown affected parasite survival in culture, viability was assessed by Hoechst staining [11]. Three day old schistosomula electroporated in the presence of a mix of SGTP1 and SGTP4 siRNAs at 2.5 µg each, were cultured in RPMI medium containing either 10 mM or 0.05 mM D-glucose for 14 days. Parasite mortality was determined in samples containing ∼100 parasites each, by adding 1 µg/ml Hoechst 33258 to the cultures at room temperature. After 10 min dead parasites were counted using a 460 nm reading filter. Viability in each group was calculated as the average value (+/− standard deviation) from triplicate experiments relative to controls treated with irrelevant siRNA.

### SGTP function: glucose uptake analysis

Schistosomula treated with siRNA specific for SGTP1, SGTP4, or a mix of equal amounts of both siRNAs, were compared for their ability to take up glucose relative to control parasites treated with an irrelevant siRNA. Schistosomula, 14 days after siRNA treatment, were washed four times in wash medium (RPMI without glucose and supplemented with 10 mM Hepes, 2 mM glutamine, and antibiotics (100 U/ml penicillin and 100 µg/ml streptomycin)) and resuspended in 30 µl of wash medium supplemented with 0.1 M D-glucose. Each sample received 1 µl of [1,2-^3^H]2-deoxyglucose at 1 µCi/ml (Amersham, Piscataway, NJ) followed by a 30 min incubation at room temperature. Parasites were subsequently washed four times in wash medium before being disrupted in 30 µl of 2% SDS. The parasite lysate was added to 1 ml Scintiverse scintillation fluid (Fischer) and subjected to liquid scintillation counting. Radiolabel uptake was calculated per 1000 schistosomula. Assays were performed in triplicate for each group and averaged for analysis. Glucose uptake in control parasites was also measured in the presence of cytochalasin B (40 µM) which was added to the parasite culture for 30 min prior to the start of the uptake experiment.

### Infection of mice with siRNA-treated schistosomula

One day old cultured schistosomula were electroporated with SGTP, or control, or no, siRNA and the groups were divided into three samples each. The first sample was immediately used to infect female BALB/c mice (∼1,000 parasites/mouse) and the other two samples were kept in culture for 7 days or for 28 days to determine the efficiency of gene knockdown (at day 7) and to monitor long-term suppression *in vitro* (at day 28). Mice were infected by injecting schistosomula in 100 µl of RPMI without phenol red into the thigh muscle of the animals using a 1 ml tuberculin syringe and a 25G-1 needle. Twenty eight days later, the mice were euthanized and adult worms recovered by portal vein perfusion. Recovered worms were counted, examined under a light microscope and subsequently their SGTP gene expression levels were determined, as described above. The same procedure was followed in experiments in which schistosomula were exposed to long dsRNA by soaking, except that mice were infected 24 h after parasite exposure to long dsRNA.

### Statistical analysis

All data were analyzed using GraphPad Prism 4 software. One Way ANOVA was used to compare median values among three or more groups. Student's *t*-tests were used to compare the means between a target group and a control group and *p* values close to or less than 0.05 were considered significant.

### Accession numbers

The following GenBank accession numbers apply to the DNAs targeted in this work: SGTP1: L25065 and SGTP4: L25067.
